# Supplementary material for: The critical role of membralin in postnatal motor neuron survival and disease
Source: eLife. 2015 May 15;4:e06500. doi: 10.7554/eLife.06500 (PMC4460860; doi:10.7554/eLife.06500)
Supplement: Supplementary file 1. — Primer list for genotyping and sequencing. DOI: http://dx.doi.org/10.7554/eLife.06500.018 [file elife06500s001.zip › elife06500s001.dot]

**Supplementary File 1 Primer list for genotyping and sequencing.**

| **Primer Name** | **Primer Sequences** |
| --- | --- |
| Mouse NR3B-WT-F | 5’ TGGGCTTGGACAGTGTGGGAAATC 3’ |
| Mouse NR3B-WT-R | 5’ TCTCTGGGTGGAACCTTTTGCTGG 3’ |
| Mouse NR3B-KO-F | 5’ TGCTCCAGACTGCCTTGGGAAAAG 3’ |
| Mouse NR3B-KO-R | 5’ ACCTCTACCACTTCGCCTTCTATGC 3’ |
| Mouse Membralin-Tg-F | 5’ GCAGAAGAACGGCATCAAGGTG 3’ |
| Mouse Membralin-Tg-R | 5’ TGGGTGCTCAGGTAGTGGTTGTC 3’ |
| Mouse Membralin-WT-F | 5’ GTTCTCGGTCACAAAGGCTAC 3’ |
| Mouse Membralin-WT-R | 5’ GTGTGGCTTGTTTATCCCAGT 3’ |
| Mouse Membralin-KO-F | 5’ GTTCTCGGTCACAAAGGCTAC 3’ |
| Mouse Membralin-KO-R | 5’ TCCTACAACACACACTCCAAC 3’ |
